# Tailoring Thermal and Electrical Properties of Jeffamine Segmented Polyetherimide Composite Films Containing BaTiO_3_ particles

**DOI:** 10.3390/polym14214715

**Published:** 2022-11-03

**Authors:** Corneliu Hamciuc, Gabriela Lisa, Diana Serbezeanu, Luiza Mădălina Grădinaru, Mihai Asăndulesa, Niță Tudorachi, Tăchiță Vlad-Bubulac

**Affiliations:** 1 “Petru Poni” Institute of Macromolecular Chemistry, Aleea Gr. Ghica Voda 41A, 700487 Iasi, Romania; 2 Department of Chemical Engineering, Faculty of Chemical Engineering and Environmental Protection, Gheorghe Asachi Technical University of Iasi, Bd. Mangeron 73, 700050 Iasi, Romania

**Keywords:** polyetherimides, Jeffamine segments, thermal decomposition mechanism, contact angle, dielectric properties

## Abstract

The continuous advancement of materials science has highlighted the ongoing need for additional studies on the main composite materials topics, particularly in the field of multifunctional nano-composites, towards improving their capability to meet multifaceted requirements in order to stimulate both scientific and technological development. In this study, we report the preparation and characterization of polyetherimides (PEIs) derived from 4,4′-(4,4′-isopropylidenediphenoxy) bis (phthalic anhydride) following a two-step polycondensation reaction using either 4,4′-(1,3-phenylenedioxy) dianiline, or Jeffamine ED-600 as comonomers, or a mixture of the two diamines. Based on the PEI containing flexible Jeffamine segments, polymer composite films were developed by incorporating BaTiO_3_ particles. The chemical structure and morphology of the composite films were investigated by FTIR spectroscopy and scanning electron microscopy. Thermal properties were determined by thermogravimetric analysis and differential scanning calorimetry. The influence of Jeffamine segments on the thermal decomposition process was investigated by TG/MS/FTIR measurements under air and nitrogen atmospheres. Based on the obtained data, the thermal decomposition mechanism was established and is discussed in accordance with the chemical structures of the polymers. The surface properties of the PEI and PEI-composite films were characterized by performing contact angle measurements. The addition of BaTiO_3_ increased the wettability of the surfaces. The dielectric characteristics of polymer composite films were investigated by broad band dielectric spectroscopy measurements. It was noticed that the addition of BaTiO_3_ nanoparticles to the copolymer matrix gradually enhanced the dielectric constant of the composites.

## 1. Introduction

Aromatic polyimides are a class of heterocyclic thermostable polymers used in many high-performance applications due to their unique characteristics such as high thermal and thermo-oxidative stability, high glass transition temperature, superior mechanical properties, good adhesion to substrate, good electrical insulating properties, and high tensile strength. These polymers are used as packaging materials, insulating layers, and polymer composite dielectrics with good thermal stability [1,2,3,4]. The disadvantages of aromatic polyimides are their infusibility and low solubility in organic solvents, which restrict their applications in many fields of industry. Much research has been undertaken to improve the processability of aromatic polyimides [5,6]. The introduction of aromatic ether linkages into the main macromolecular chains considerably improves the solubility of polyimides without largely affecting their thermal stability. Thus, aromatic polyetherimides (PEIs), which result from the reaction of bisetheranhydrides and aromatic diamines, are considered high-performance engineering thermoplastics, having excellent thermal stability provided by the presence of imide rings and good processability due to the presence of aromatic ether linkages [7]. In addition to high thermal stability, PEIs have high tensile strength, good electrical properties, UV-light resistance, hydrolytic stability, good flame resistance, and low smoke emission.

The preparation of segmented copolymers is a powerful method to produce new polymer materials with desired properties. For example, the introduction of flexible segments coming from Jeffamine monomers in the macromolecular chains of polyimides improves their characteristics: it increases the dielectric constant, improves the biocompatibility, and leads to materials more suitable for gas separation membranes. The Jeffamine monomer has two reactive amine groups attached at the chain ends of poly (propyleneoxide) (PPO), poly (ethylene oxide) (PEO), or mixed PPO/PEO building blocks. Polyimides and PEIs containing Jeffamine segments have been prepared and characterized. They have potential applications as materials with high dielectric constant [8], membranes for gas separation [9,10], hydrophilic polyimides for microfiltration [11], materials for lithium polymer batteries [12], or materials for biomedical applications [13,14]. The thermal stability and toxicity of the volatiles eliminated during thermal decomposition of polymeric materials are very important because they dictate the possible applications and can give an indication of the influence they have on the environment in case of fire. The thermal degradation of polyethylene glycol (PEG) in nitrogen atmosphere was studied by different techniques and the main decomposition products were reported. One of the products was found to be formaldehyde, a carcinogen product [15]. A radical mechanism was proposed for the thermal decomposition of PEG in nitrogen atmosphere. It involved homolytic cleavage, disproportionation, and radical abstraction [15,16,17]. Previously, we studied thermal and thermo-oxidative stability, and the degradation mechanism of some heterocyclic polymers, by simultaneously using mass spectrometry and Fourier transform infrared spectroscopy of off-gases (TG/MS/FTIR analysis) from a thermogravimetric analyzer in an air and inert atmosphere [18,19,20,21]. Thus, in the case of some PEIs that were studied, it was found that the main products of thermal decomposition in the air atmosphere were water, carbon dioxide, carbon monoxide, benzene, methane, and formaldehyde, while in an inert atmosphere, phenol and benzonitrile were also observed [22]. The mechanism of thermal decomposition in air or inert atmosphere of some PEIs containing polydimethylsiloxane (PDMS) segments was also studied [20]. In this case, the thermal decomposition started with the decomposition of PDMS segments with the formation of some cyclic structures (hexamethylcyclotrisiloxane). One way to enhance the dielectric properties of a polymer consists of the incorporation of an inorganic filler with a high dielectric constant.

Recent research has focused on the impact of incorporating inorganic fillers into various high-performance polymer composites [23,24,25]. Barium titanate (BaTiO_3_) is an inorganic filler of interest due to its high dielectric constant and ferroelectric properties. Polyimide nanocomposites containing BaTiO_3_ nanoparticles have been reported in the literature. C.Y. Lin et al. reported the preparation of organo-soluble polyimides/BaTiO_3_ composites having a dielectric constant of 52 at 50 vol % filler [21]. S. Yue et al. obtained polyimide composite films with reduced BaTiO_3_ having enhanced dielectric constant, low dielectric loss, and energy storage properties [22]. Z.M. Dang et al. prepared BaTiO_3_/polyimide nanocomposite films with high dielectric permittivity (20), high breakdown strength (67 MV/m), and high thermal stability [26]. Polyimide composites containing 50 vol % BaTiO_3_ that exhibited a dielectric constant of 35 and a dielectric loss tangent of 0.0082 at 10 kHz were also described in the literature [27].

In this paper, composite polymer films containing BaTiO_3_ were prepared using a PEI containing segments from a Jeffamine monomer, as polymer matrix. The structural, morphological, thermal, and electrical characterization of the films was carried out. The study of the thermal decomposition mechanism of PEI containing Jeffamine segments was performed based on TG/MS/FTIR analysis.

## 2. Materials and Methods

### 2.1. Materials

4,4′-(4,4′-Isopropylidenediphenoxy)bis (phthalic anhydride)-97% (6HDA, Molecular weight: 520.49 g/mol), 4,4′-(1,3-phenylenedioxy)dianiline-98% (Molecular weight: 292.33 g/mol), O,O′-bis (2-aminopropyl)polypropylene glycol-block-polyethylene glycol-block-polypropylene glycol) (Jeffamine ED-600, 600 MW, 30 mequiv NH_2_/g), *N*-methyl-2-pyrrolidone-99.5% (NMP), BaTiO_3_ nanoparticles (cubic crystalline phase, <100 nm particle size (BET)) were provided by Sigma-Aldrich (Steinheim, Germany) and used as received.

### 2.2. Synthesis of PEIs (1–3)

**Scheme 1:**
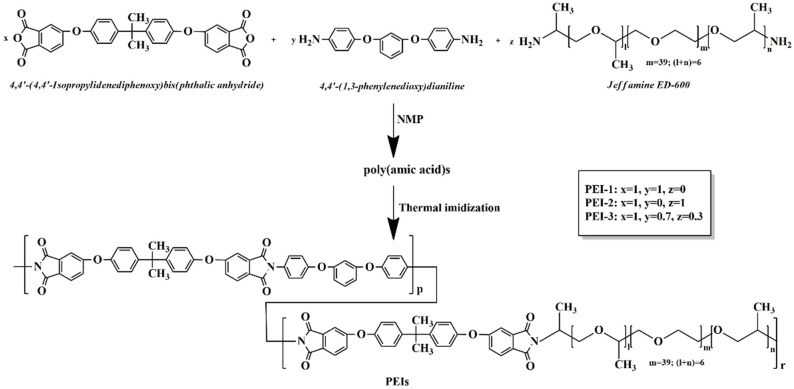
Preparation of PEI (1–3).

PEIs (1–3) were synthesized through a two-stage procedure. In the first stage, poly (amic acid)s were prepared by the polycondensation reaction of mixtures of two diamines, 4,4′-(1,3-phenylenedioxy)dianiline and Jeffamine ED-600 (in three molar ratios: 0:1 (PEI-1); 1:0 (PEI-2), and 0.7:0.3 (PEI-3) with 6HDA (Scheme 1). An example is given for the preparation of polymer PEI-3: 4,4′-(1,3-phenylenedioxy)dianiline (2.04 g, 7 mmol), Jeffamine ED-600 (1.56 g, 3 mmol), and NMP (35 mL) were introduced into a 100 mL three-necked flask equipped with a magnetic stirrer and nitrogen inlet and outlet. The mixture was stirred under a nitrogen atmosphere to complete dissolution. Then, 6HDA (5.20 g, 10 mmol) was added and stirring was continued for 12 h. In the second stage, the solution of poly (amic acid) PEI-3′ was cast onto glass plates and heated at 75, 100, 125, 150 m and 200 °C, for one hour at each temperature. The polymer film PEI-2 was thermally treated up to 250 °C. The PEI-2 and PEI-3 films were taken off the glass plates by immersion in hot water. PEI-1 gave only coatings, and no flexible films could be detached from the glass plates.

### 2.3. Preparation of the Composites PEI-3 (10–30%)

BaTiO_3_ nanoparticles were homogeneously dispersed in NMP, in a round flask, by sonicating the resulting mixture for one hour, and then, by magnetic stirring for an additional 5 h. Then, a mixture of Jeffamine ED-600 and 4,4′-(1,3-phenylenedioxy) dianiline (molar ratio: 0.3:0.7) was introduced into the flask and, after the complete dissolution of these two components, 6HDA was added. The reaction was continued under stirring for 12 h; the resulting homogeneous suspension was cast onto glass plates which were subjected to a thermal treatment by heating at 75, 100, 125, 150, and 200 °C, for one hour at each temperature, with the aim of obtaining the corresponding fully cyclized PEI-3 composites in the film form. Subsequently, the polymer films were purified by Soxhlet extraction in ethanol, to remove unreacted monomers and hardly volatile residual solvent. Three composite films, PEI-3-10%, PEI-3-0%, and PEI-3-30%, having theoretically 10, 20, or 30 wt% BaTiO_3_, respectively, were prepared.

### 2.4. Measurements

FTIR spectra of the composite PEI (1–3) and PEI-3 (10–30%) were obtained using a BioRad ‘FTS 135’ FTIR spectrometer equipped with a Specac “Golden Gate” ATR accessory. A LUMOS Microscope Fourier Transform Infrared (FTIR) spectrophotometer (Bruker Optik GmbH, Ettlingen, Germany), equipped with an attenuated total reflection (ATR) device was used to record the scans between 4000 and 500 cm^−1^ at a resolution of 4 cm^−1^.

Differential scanning calorimetry (DSC) measurements on composite PEIs (1–3) and PEI-3 (10–30%) were carried out using a Mettler Toledo DSC1 type device (Netzsch, Germany) in an inert atmosphere, with a heating rate of 10 °C/min.

The coupled technique TG/MS/FTIR was investigated using a device consisting of a thermogravimetric analysis device type STA 449 F1 Jupiter (Netzsch, Germany), coupled with a spectrophotometer type Vertex-70 FTIR (Bruker, Germany) and a mass spectrometer QMS model 403 C Aëolos (Netzsch, Germany). The mass of PEI (1–3) and PEI-3 (10–30%) ranged between 7.3 and 8.4 mg, and the applied heating rate was 10 °C/min with a flow of 50 mL/min. Further information on the technical characteristics and operation of the TG/MS/FTIR system is detailed in a previous paper [18].

Microscopic investigations of composite films were performed on an Verios G4 UC Scanning Electron Microscope (Thermo Scientific, Brno, Czech Republic), operating at 10 kV with secondary electrons in low-vacuum mode (LFD detector). The Verios G4 UC Scanning Electron Microscope is equipped with an Energy Dispersive X-ray (EDX) system for qualitative and quantitative analysis and elemental mapping.

The wettability of the surfaces of the PEI (1–3) and PEI-3 (10–30%) was evaluated by static contact angle measurements using the sessile drop method at room temperature. The measurements were carried out using a CAM 101 Optical Contact Angle Instrument (KSV Instruments, Helsinki, Finland) equipped with a special optical system connected to a computer. Approximately 1 μL of tested liquids (water (W) and ethylene glycol (EG)) were placed with a Hamilton syringe on the sample surface and the images were sent via the CCD camera to the computer for analysis. Ten photos were recorded at an interval of 0.016 s. All the measurements were done in triplicate and the results were recorded as mean ± standard deviation. The surface parameters (adhesion work, surface free energy, interfacial solid–liquid tension) were estimated based on contact angle values and the following equations developed by Owens–Wendt–Rabel–Kaelbe [28]:

(1) $$W_{a\;}=\gamma_{LV}+\gamma_{SV}-\gamma_{SL}=\gamma_{LV}\left(1+\cos\theta \right)$$

(2) $$\;\gamma_{SV}=\gamma_{SV}^{p}+\gamma_{SV}^{d}$$

(3) $$\gamma_{LV}\left(1+\cos\theta \right)=2\sqrt{\gamma_{SV}^{p}\gamma_{LV}^{p}}+2\sqrt{\gamma_{SV}^{d}\gamma_{LV}^{d}}$$

(4) $$\gamma_{SL}=\gamma_{SV}+\gamma_{LV}-2\left(\sqrt{\gamma_{SV}^{p}\gamma_{LV}^{p}}+\sqrt{\gamma_{SV}^{d}\gamma_{LV}^{d}}\right)$$

where *W_a_* is the adhesion work, *γ_LV_* is the liquid–vapor surface tension, *γ_SV_* is the energy of surface, *γ_SL_* is the solid drop interfacial tension, cos *θ* is the drop–surface contact angle, *γ^p^_SV_* is the polar component of the surface tension, which includes two types of coulomb interactions, dipole–dipole and dipole-induced dipole, and *γ^d^_SV_* is the dispersive component of the surface tension, which represents van der Waals interactions. The surface tension values for the two liquids and their components (mN/m) are *γ_LV_* = 72.8, *γ^p^_LV_* = 51, *γ^d^_LV_* = 21.8 for water and *γ_LV_* = 48, *γ^p^_LV_* = 19, *γ^d^_LV_* = 29 for ethylene glycol, respectively.

Broadband dielectric spectroscopy measurements were carried out on a dielectric spectrometer (Novocontrol Technologies, Montabaur, Germany) in a frequency window between 1 Hz and 1 MHz and at temperatures between −120 and 170 °C. The free-standing films were sandwiched between two gold-coated electrodes and then placed in the BDS active cell. Measurements were carried out in a dry nitrogen atmosphere, avoiding the moisture absorption.

## 3. Results and Discussion

### 3.1. Structural and Morphological Characterization of PEIs (1–3) and PEI-3 (10–30%)

**Figure 1:**
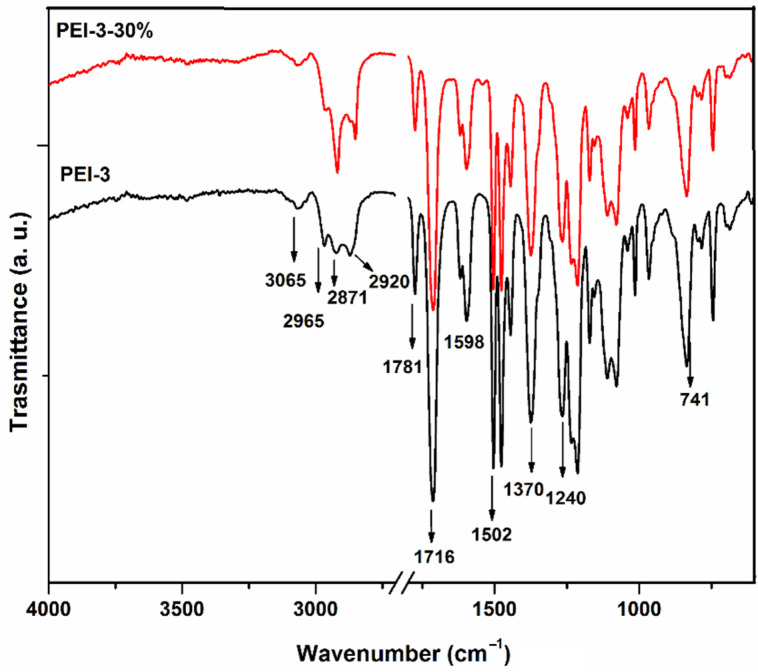
FTIR spectra of PEI-3 and PEI-3-30%.

The structures of the polymers PEIs (1–3) and PEI-3 (10–30%) were investigated by FTIR spectroscopy and ^1^H NMR (Figure S1 Supplementary Materials). In the FTIR spectra of the polymers (Figure 1 for PEI-3) characteristic absorption bands for imide rings appeared at 1780 and 1725 cm^−1^ (asymmetric and symmetric stretching vibration of C=O of imide rings), and 1365 cm^−1^ (C–N stretching vibration of imide ring) and 725 cm^−1^ (imide ring deformation). Absorption bands also appeared at 2971, 2920, and 2860 cm^−1^ (C–H aliphatic), 3060 cm^−1^ (C–H aromatic), 1598 and 1502 cm^−1^ (–C=C– aromatic), and 1240 cm^−1^ (C–O–C aromatic ether).

The polymers PEIs (1–3) were soluble in polar aprotic solvents such as DMF or N,N-dimethylacetamide, and in less polar solvents such as tetrahydrofuran or chloroform. The presence of aromatic ether linkages, doubled by the existence of flexible Jeffamine segments in the polymer structure, increases the flexibility of the macromolecules, thus improving the solubility.

**Figure 2:**
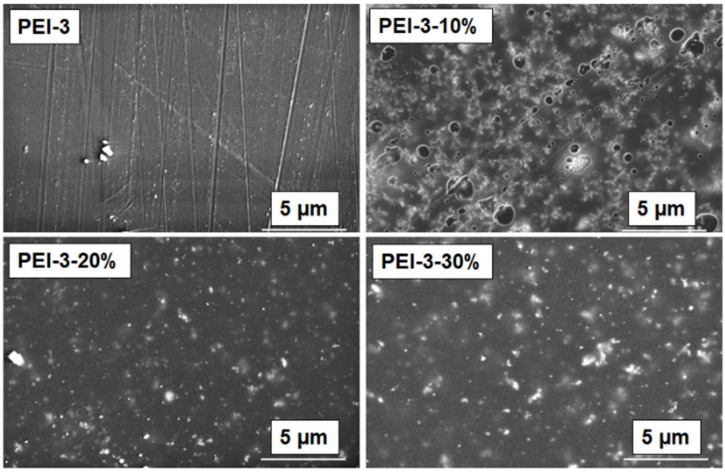
SEM micrographs of PEI-3 film and PEI-3 (10–30%) composite films.

The presence of BaTiO_3_ in the structure of PEI-3 was confirmed by the SEM investigation. From Figure 2 and Figure S2a–d Supplementary Materials, uniform distribution of the BaTiO_3_ in the PEI-3, can be observed. Pure PEI-3 film had a smooth surface and laminated structure (Figure 2). The morphological changes induced by the presence of BaTiO_3_ filler in PEI-3 (10–30%) are clearly observed from Figure 2. It can be seen that the compact morphology of the PEI-3 film filled with BaTiO_3_ powder increases with the particle content from 10 to 30 wt%. Moreover, in all cases, there are large aggregates in PEI-3 film.

### 3.2. Thermal Properties

#### 3.2.1. Influence of the Test Atmosphere on Thermal Stability of PEIs (1–3) and PEI-3-20% BaTiO_3_ Composite

Thermogravimetric (TG) and derived thermogravimetric (DTG) curves were recorded in two test atmospheres, air and nitrogen (Table 1, Figures S3a,b and S4a,b Supplementary Materials). A complex degradation mechanism was found that included two or three processes in an inert atmosphere and three or four processes in air depending on the composition of these polymers. The obtained results revealed the fact that regardless of the test atmosphere, the fully aromatic polyimide PEI-2 had the best thermal stability. This sample had the highest thermal decomposition onset temperature and the highest amount of residue, approximately 54 wt% in nitrogen and 52 wt% in air, at the end of the respective 650 °C (Table 1). Samples containing Jeffamine segments had lower thermal stability, the onset of thermal decomposition being at approximately 400 °C in nitrogen and at approximately 378 °C in air.

The amount of residue remaining in an inert atmosphere at a temperature of 650 °C for the composite containing 20 wt% BaTiO_3_ was 10% higher than that obtained for the base polymer. The amount of residue obtained at the end of the test was lower in air than in nitrogen for all samples, proving lower thermal stability in oxidative atmosphere.

The DSC curves obtained for the second heating in the temperature range of 40–175 °C indicated a glass transition temperature (*T_g_*) at 158 °C for the aromatic polyetherimide PEI-2 (Figure S5 Supplementary Materials). For the copolymer PEI-3 containing Jeffamine segments, the glass transition temperature was 100 °C, while for the composite containing 20 wt% BaTiO_3_, *T_g_* was 96 °C.

#### 3.2.2. Influence of the Test Atmosphere on the Degradation Mechanism of the PEIs (1–3) and PEI-3-20%

Studying the degradation mechanisms and the influence of the test atmosphere is an important aspect in the characterization of new materials because it can contribute to the development of materials with greater resistance and lifetime. The TG/MS/FTIR technique applied to different types of materials also contributes to finding solutions to increase the degradation rate in the case of high-volume polymers, which is important for the material recycling industry.

**Figure 3:**
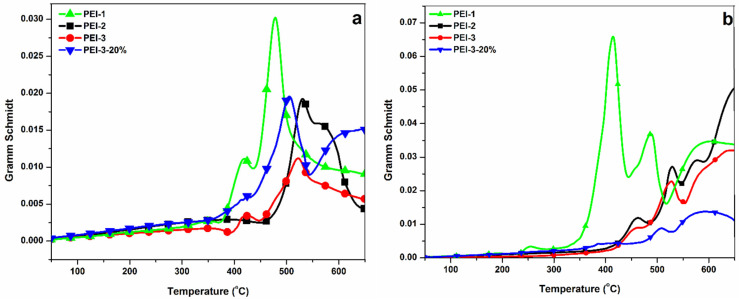
Gram–Schmidt diagram in nitrogen (**a**) and air (**b**) for PEIs (1–3) and PEI-3-20%.

According to the information presented in the previous section for all the analyzed polymers, it was found that the thermal decomposition is influenced by the test atmosphere. That is why the TG/MS/FTIR technique was applied both in air and in nitrogen to highlight the differences between the thermal decomposition mechanisms in the two test atmospheres. The Gram–Schmidt curves of total FTIR absorbance intensity shown in Figure 3a,b indicate a higher total absorbance intensity when the working atmosphere was air, confirming higher percentage mass losses in air atmosphere. Gram–Schmidt curves highlight the differences that appear in the sequence and intensity of decomposition processes depending on the test atmosphere.

In the inert atmosphere, in the graphs from Figure 3a, a peak of the total absorption intensity for the samples containing Jeffamine segments, PEI-1, PEI-3 and PEI-3-20%, was observed at approximately the same temperature (428 °C), indicating the same mechanism of thermal decomposition initiation for these samples. This peak was also found at the same temperature in air atmosphere with the same intensity for PEI-3-20%. A peak appeared at 421 °C, with a much higher intensity for PEI-1 in air atmosphere.

**Figure 4:**
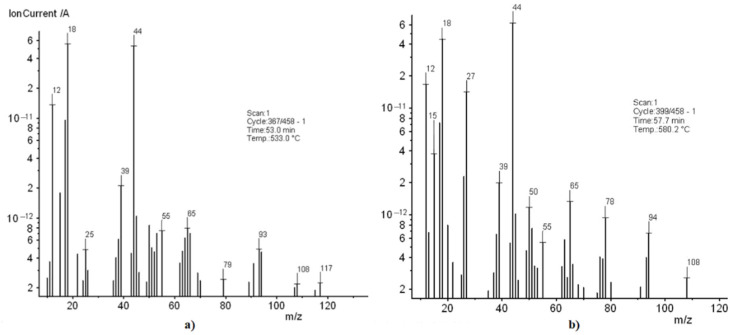
MS spectra for thermal degradation of PEI-2 in nitrogen at 533 °C (**a**) and 580 °C (**b**).

**Figure 5:**
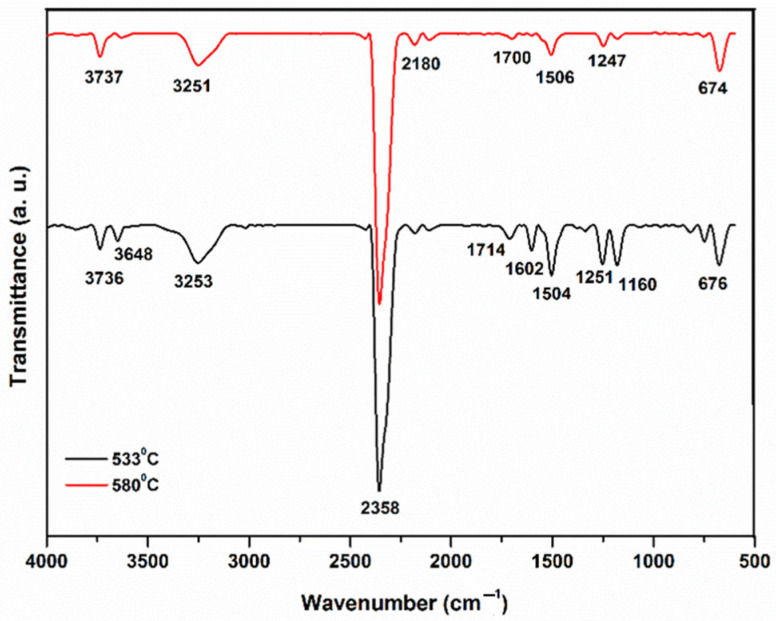
FTIR spectra for PEI-2 at various temperatures for thermal decomposition in nitrogen.

For PEI-2, according to the obtained MS and FTIR spectra, the initiation of thermal decomposition in nitrogen occurred at the bisphenol A group. The ionic fragments with the highest intensity in the first decomposition process according to Figure 4a were for m/z = 18 (H_2_O^+^), m/z = 44 (CO_2_^+^) (Figure S6 Supplementary Materials), and m/z = 39 (C_3_H_3_^+^). The fragment m/z = 117 (C_6_H_5_–CH=CH–CH_3_^+^) appeared with lower intensity, which confirms the initiation of thermal decomposition at the bisphenol A group [20]. According to the mass spectrum in Figure 4b, the ionic fragment was highlighted in the second decomposition process m/z = 27 (HCN^+^), m/z = 78 (C_6_H_5_^+^) (Figure S7 Supplementary Materials), m/z = 94 (C_6_H_5_OH^+^) (Figure S7 Supplementary Materials), and m/z = 103 (C_6_H_5_CN^+^) (Figure S7 Supplementary Materials), which suggests the onset of degradation of the polyimide group. The presence of the ionic fragment m/z = 108 (HO–C_6_H_4_–OH^+^) (Figure S7 Supplementary Materials) was also highlighted, which indicates the thermal decomposition of the 1,3-phenylenedioxy group. These results were also confirmed by the FTIR spectra for the gas phase resulting from the thermal decomposition of polymer PEI-2 in an inert atmosphere, shown in Figure 5. The characteristic bands for water were present in the range of 4000–3400 cm^−1^, for CO_2_ in the range of 2400–2200 cm^−1^ and at 740–600 cm^−1^, for benzene in the range of 1600–1400 cm^−1^ [20], and for HCN at 1300–1100 cm^−1^ [29].

**Figure 6:**
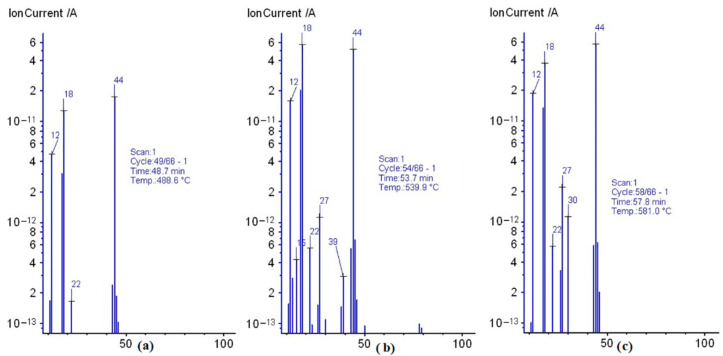
MS spectra for PEI-2 degradation in air at 489 °C (**a**), 540 °C (**b**), and 581 °C (**c**).

When the thermal decomposition of PEI-2 took place in air, no ionic fragments with m/z > 100 were identified according to the MS spectra in Figure 6. Only the ionic fragments m/z = 18 (H_2_O^+^) and m/z = 44 (CO_2_^+^) were observed at the temperature of 489 °C (Figure S9 Supplementary Materials), whose intensity increased at the temperature of 540 °C when other ionic fragments (m/z = 15 (CH_3_^+^) m/z = 27 (HCN^+^), m/z = 39 (C_3_H_3_^+^), and m/z = 78 (C_6_H_5_^+^)) were identified (Figure S10 Supplementary Materials). The ionic fragment m/z = 30 (CH_2_O^+^) (Figure S9 Supplementary Materials) was also observed, whose intensity increases at the temperature of 581 °C.

**Figure 7:**
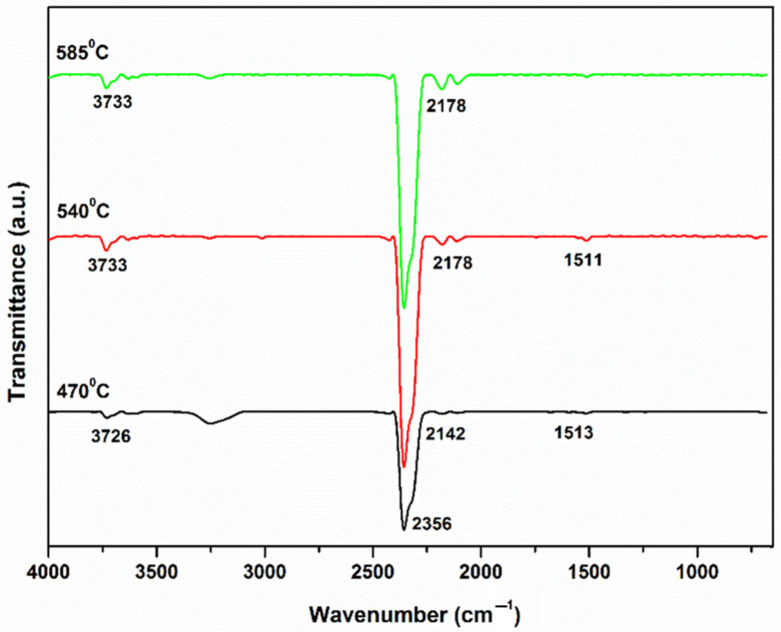
FTIR spectra for PEI-2 at various temperatures for thermal decomposition in air.

**Scheme 2:**
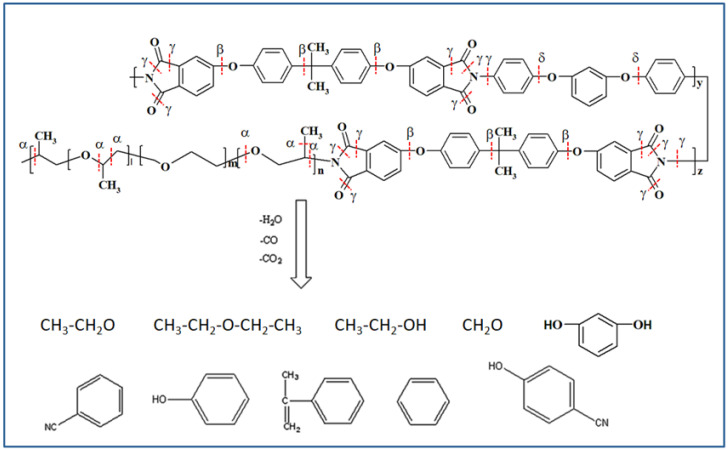
The proposed thermal degradation mechanism of PEIs (1–3).

The FTIR spectra represented in Figure 7 were in full agreement with the MS results. The characteristic peaks were observed for water in the range 4000–3400 cm^−1^, for CO_2_ in the range 2400–2200 cm^−1^, and for benzene in the range 1600–1400 cm^−1^. In the case of samples containing Jeffamine segments, thermal decomposition in an inert atmosphere began at a temperature of approximately 400 °C, with the fragmentation of the polypropyleneglycol-block and polyethyleneglycol-block (Scheme 2). The variation in the ionic current (Figure S11 Supplementary Materials) for the ionic fragments of PEI-1, PEI-3, and PEI-3-20% was found to be m/z = 58 (CH_3_CH_2_CHO^+^), m/z = 73 (CH_3_CH_2_OCH_2_CH_3_^+^), m/z = 15 (CH_3_^+^), respectively, and m/z = 46 (C_2_H_5_OH^+^) in the temperature range of 400–510 °C. In the same temperature range, the variation in the ionic current also appeared for the fragments: m/z = 18 (H_2_O^+^), m/z = 28 (CO^+^), m/z = 44 (CO_2_^+^), and m/z = 30 (CH_2_O^+^), as presented in Figure S6. The decomposition of the bisphenol A group, the polyimide group, and the 1,3-phenylenedioxy group began at temperatures higher than 510 °C. Figure S7 shows the variation in the ionic current for some of the ionic fragments resulting from their decomposition, respectively, m/z = 78 (C_6_H_5_^+^), m/z = 91 (C_7_H_7_^+^), m/z = 94 (C_6_H_5_OH^+^), and m/z = 108 (HOC_6_H_4_OH^+^). As can be seen from Figure S7, the fragment m/z = 94 was not present in the case of PEI-3-20%, and the fragment m/z = 108 in the case of PEI-1. In the case of PEI-3-20%, due to the presence of oxygen in a larger amount, a higher intensity of the peaks corresponding to the fragment m/z = 18 (H_2_O^+^) and fragment m/z = 44 (CO_2_^+^) (Figure S6 Supplementary Materials) was found. The lack of ion current variation for the m/z = 108 fragment in the case of PEI-1 was related to the fact that it does not contain the 1,3-phenylenedioxy group. According to Figure S8 from the Supplementary Materials, the presence of the fragment m/z = 103 (C_6_H_5_CN^+^) was highlighted in the case of all samples, which can also result in the ionic fragment m/z = 44 (CO_2_^+^) (Figure S6) and the fragment m/z = 28 (CO^+^) (Figure S6) from the degradation of the polyimide group [7,20,30,31].

**Figure 8:**
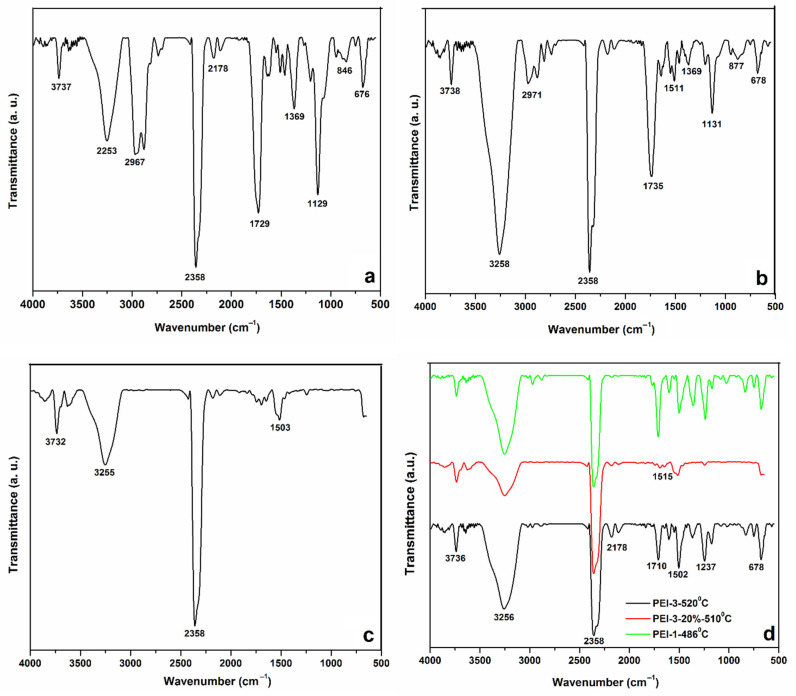
FTIR spectra for PEI-1 at 426 °C (**a**), PEI-3 at 427 °C (**b**), and PEI-3-20% at 429 °C (**c**), and these samples at higher temperatures for thermal decomposition in nitrogen (**d**).

The thermal decomposition initiation mechanism for PEI-1, PEI-3, and PEI-3-20% presented in Scheme 2 was also supported by the FTIR spectra recorded for the gas phase resulting from the decomposition of these polymers at a temperature of approximately 428 °C, as shown in Figure 8a–c. The presence of bands characteristic of aliphatic groups –CH_3_, –CH_2_– in the area 3100–2600 cm^−1^, of etheric groups C–O–C with a peak at approximately 1250 cm^−1^, of carbonyl groups C=O (aldehydes) at approximately 1730 cm^−1^, and hydroxyl groups in the area 4000–3100 cm^−1^ was found, as shown in Figure 8 [32]. At higher temperatures, according to the spectra shown in Figure 8d, the intensification of the characteristic bands for CO_2_ was observed in the range 2400–2200 cm^−1^ and at 740–600 cm^−1^, for benzene in the range 1600–1400 cm^−1^ [20], and for CO in the range 2200–2000 cm^−1^. There was also a peak at 1367 cm^−1^ which can be associated with the presence of benzonitrile [33].

**Figure 9:**
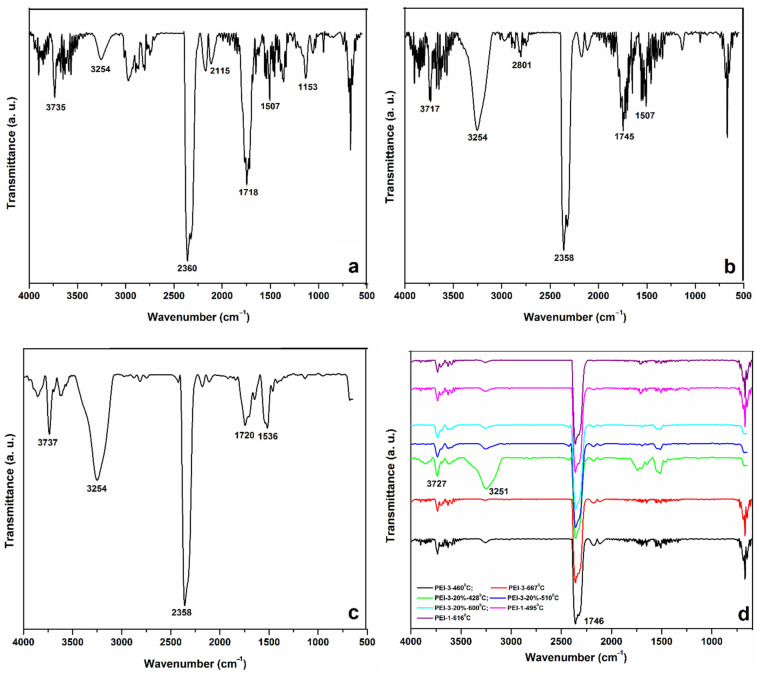
FTIR spectra for PEI-1 at 426 °C (**a**), PEI-3 at 415 °C (**b**), and PEI-3-20% at 395 °C (**c**), and PEI-1, PEI-3 and PEI-3-20% at various temperatures for decomposition in air (**d**).

The analysis of the thermal decomposition process in air atmosphere of samples containing Jeffamine segments (PEI-1, PEI-3, and PEI-3-20%) revealed that the thermal decomposition initiation mechanism was similar to the one previously described in nitrogen atmosphere (Scheme 2). The ionic fragments (Figure S12 Supplementary Materials) were found in the mass spectra: m/z = 58 (CH_3_CH_2_CHO^+^), m/z = 73 (CH_3_CH_2_OCH_2_CH_3_^+^), m/z = 15 (CH_3_^+^), respectively, and m/z = 46 (C_2_H_5_OH^+^), whose variation began at a temperature of approximately 360 °C, lower than in the case of decomposition in a nitrogen atmosphere. The variation in the ionic current also began at approximately the same temperature for the fragments: m/z = 18 (H_2_O^+^), m/z = 28 (CO^+^), m/z = 44 (CO_2_^+^), and m/z = 30 (CH_2_O+) as presented in Figure S9. Figure S10 shows the variation in the ionic current for the ionic fragments: m/z = 78 (C_6_H_5_^+^), m/z = 91 (C_7_H_7_^+^), m/z = 94 (C_6_H_5_OH^+^), and m/z = 108 (HOC_6_H_4_OH^+^). The fragment m/z = 108 showed no variation for any of the samples, the fragment m/z = 94 appeared only in the case of PEI-1, and the fragment m/z = 91 showed variation only in the case of PEI-1 and PEI-3. These observations indicated that the thermal decomposition of the bisphenol A group and the 1,3-phenylenedioxy group was influenced by the test atmosphere, an aspect also confirmed in previous studies [7,20,34]. The decomposition mechanism of the polyimide group was not influenced by the atmosphere in which the thermal decomposition took place. Figure S13 shows the variation of the fragment m/z = 103 (C_6_H_5_CN^+^), which can result from the degradation of the polyimide group alongside the ionic fragment m/z = 44 (CO_2_^+^) (Figure S9) and the fragment m/z = 28 (CO^+^) (Figure S9) [7,20,30,31]. The observations obtained from the analysis of the MS spectra for decomposition in air are also supported by the FTIR spectra shown in Figure 9.

Figure 9a–c shows the presence of bands characteristic of aliphatic groups –CH_3_, –CH_2_– in the 3100–2600 cm^−1^ area, which were more intense in the case of PEI-1, of C–O–C etheric groups with a peak at approximately 1250 cm^−1^, of carbonyl groups C=O (aldehydes) at approximately 1730 cm^−1^, and of hydroxyl groups in the area 4000–3100 cm^−1^ [32]. The intensification of the characteristic CO_2_ bands was observed at higher temperatures in the range 2400–2200 cm^−1^ and at 740–600 cm^−1^, according to the spectra presented in Figure 9d. The characteristic bands were highlighted for benzene in the range 1600–1400 cm^−1^ [20] and for CO in the range 2200–2000 cm^−1^. We also observed a lower intensity peak at 1367 cm^−1^, which may be associated with the presence of benzonitrile [33]. The proposed decomposition mechanism for Jeffamine is also confirmed by other existing studies in the literature. Thus, Song and others identified using the gas chromatography technique coupled to time-of-flight mass spectrometry (GC-TOF-MS) that the majority product of the decomposition of propylene glycol is the fragment C_3_H_7_O^+^ [35]. Voorhees et al. evaluated the decomposition mechanism of polyethylene glycol under nitrogen atmosphere at a heating rate of 20 °C/min and identified volatile products with less than four atoms using gas chromatography-mass spectrometry (GC/MS) (formaldehyde, ethanol, ethylene oxide, etc.), but also carbon dioxide, water etc. [36]. The fragment m/z = 73 (CH_3_CH_2_OCH_2_CH_3_^+^) was also identified by Hiltz in a pyrolysis-gas chromatography/mass spectrometry (py-GC/MS) study of polyethylene glycol [37]. In the case of thermal decomposition of polyethylene glycol in air, *α*-hydroperoxide is formed, which is thermally labile and fragments itself through a radical mechanism, and, in the end, results compounds containing carbonyl group [38]. Kitahara and Fujii analyzed the thermal decomposition of polyethylene glycol using a technique combining evolved gas analysis (time-resolved pyrolysis) with ion-attachment mass spectrometry. Formaldehyde, a product that is carcinogenic, was identified among the 10 most abundant decomposition products. Kitahara and Fujii pointed out that a series of highly reactive organic peroxides such as CH_3_OOH and HOCH_2_OOH result from the decomposition of polyethylene glycol in air and cannot be identified by other techniques [15]. Finally, these products lead to obtaining formaldehyde. Our study found a fourfold increase in the ionic current strength for this fragment if the decomposition occurs in air in comparison to nitrogen.

### 3.3. Wettability and Determination of Surface Energy Parameters

Surface wettability is a physical characteristic that controls the interaction between the solid and liquid phases and is important in various biological systems and technological applications. In order to evaluate the surface wettability, the contact angle measurements were performed using two different liquids: water and ethylene glycol. The obtained values are shown in Table 2. The introduction of Jeffamine monomer considerably decreased the water contact angle value from 61.78° for PEI-2 to 49.79° for PEI-3 and, therefore, PEI-3 has better wettability. In contrast, when EG was used as the polar test liquid, the contact angle value increased, due to the lower value of *γ_LV_* of EG. A lower value of contact angle indicates better adhesion of liquids on the surfaces. The addition of BaTiO_3_ increased the wettability of the surfaces up to 13° for PEI-3-30%. The strength of binding between the phases (liquid–solid) could be assessed by determining the work of adhesion (*W_a_*), which is dependent on the surface tensions of the tested liquids and their contact angles on the surfaces. The calculated values are illustrated in Table 2.

Generally, the work of adhesion is typically high and more energy is required to separate the solid from the liquid when the contact angle value is small [28]. The values of *Wa* increased with the decrease in the contact angle values. Moreover, *Wa* measured using more polar liquid (W) was higher than measured using less polar liquid (EG) for all values of s, due to the strong interactions between these liquids and PEI surfaces. The surface properties of solids, which are reflected in surface free energy, are the key to understanding the mechanism of surface phenomena, which in turn, is crucial in various applications such as adhesion, coating, and printing. To determine the surface parameters (*γ_SV_*, *γ^p^_SV_*, *γ^d^_SV_*, *γ_SL_*), the measured contact angles were inserted into the mathematical equations formulated by Owens, Wendt, Rabel, and Kaelble [28].

The results illustrated in Table 2 indicate a significant difference between the studied samples. Firstly, the addition of Jeffamine monomer increased the value of *γ_SV_* from 39.48 mN/m (PEI-2) to 61.42 mN/m (PEI-3). Moreover, when BaTiO_3_ was added, *γ_SV_* increased to 92.17 mN/m (PEI-3-30%). Secondly, the results indicated a significant difference between the polar (*γ^p^_SV_*) and the dispersive (*γ^d^_SV_*) components; the polar contributions exceeded the dispersive components in the majority of the sample, confirming the hydrophilic character of the surfaces. Thus, when comparing PEI-2 and PEI-3, the difference between the surface components was higher for PEI-3, due to the addition of Jeffamine monomer, which increased more the polar interactions such as hydrogen bonds or dipole forces. However, by increasing the BaTiO_3_ content, a substantial increase of γ^p^_SV_ and a decrease of γ^d^_SV_ appeared, especially in the case of PEI-3-30%. The interfacial solid–liquid tension (γ_SL_) values increased with decreasing contact angle values. These values can be high or low, depending on the attractive forces between the molecules of the liquid and solid. As a result, if the attraction between the liquids and surface is high, then the interfacial tension is high and the liquid adheres to the surface more easily. In conclusion, the wettability of these composites increased as a result of the presence of more polar groups at the surfaces, due to the incorporation of Jeffamine monomer and addition of BaTiO_3_.

### 3.4. Dielectric Spectroscopy Measurements

**Figure 10:**
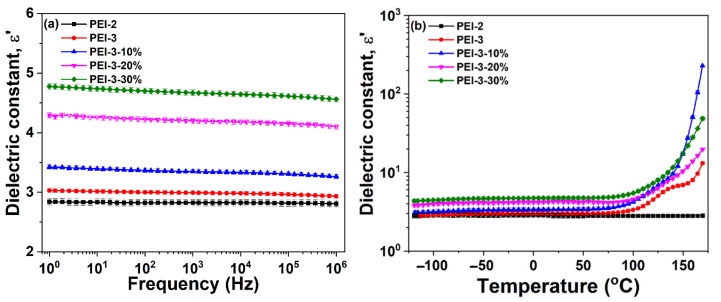
The evolution of dielectric constant with frequency at room temperature (**a**) and with temperature at 10 Hz (**b**) for the studied samples.

The behavior of the dielectric constant, *ε*′, as a function of frequency and temperature, is shown in Figure 10a,b, respectively. Following Figure 10a, the dielectric constant, *ε*′, had a slight decrease with increasing frequency, as generally observed for PEI-based materials [39]. The smallest magnitude of *ε*′ was observed for the fully aromatic polyimide PEI-2, revealing a low dipolar activity (e.g., *f* = 10 Hz, *ε*′ = 2.8). The incorporation of the Jeffamine segments increased the *ε*′ magnitude of PEI-3 (e.g., at *f* = 10 Hz, *ε*′ = 3). It is worth noting that the addition of BaTiO_3_ nanoparticles in the copolymer matrix gradually enhanced the dielectric constant of the composites (e.g., at *f* = 10 Hz, for the composite with 10% fraction of BaTiO_3_, *ε*′ = 3.4, while for the composite with 30% BaTiO_3_ content, *ε*′ = 4.7). From the isochronal version of *ε*′ vs. temperature, retrieved in Figure 10b, we emphasize the thermal stability of the dielectric constant for the entire temperature range. On the other hand, *ε*′ of Jeffamine-based copolymers was constant only at temperatures between −120 and 90 °C. Above the *Tg* of copolymers, the cooperative motions of chain segments from the macromolecule backbone were activated and, consequently, the magnitude of *ε*′ increases dramatically.

**Figure 11:**
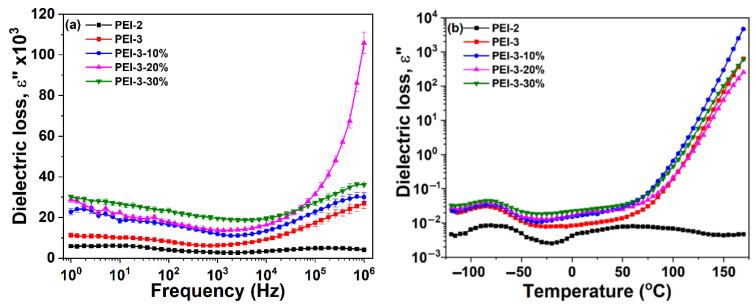
The evolution of dielectric loss with frequency at room temperature (**a**) and with temperature at 10 Hz (**b**) for the studied materials.

The dielectric loss of copolymers, *ε*″, was low, <0.01 and generally increased with the addition of BaTiO_3_ fraction (Figure 11a). Particularly for PEI-3-20%, the intense signal detected in the high frequency region was due to a possible electrode polarization effect that appeared during the experimental dielectric measurements. The temperature dependences of *ε*″ reveals the presence of the secondary relaxation dipolar processes: the *γ*-relaxation at temperatures between −120 and −20 °C, and the *β*-relaxation at temperatures between −20 and 150 °C. In addition, at temperatures higher than 50 °C, *ε*″ of Jeffamine-based copolymers is intensively enlarged, probably due to the presence of charge carriers.

**Figure 12:**
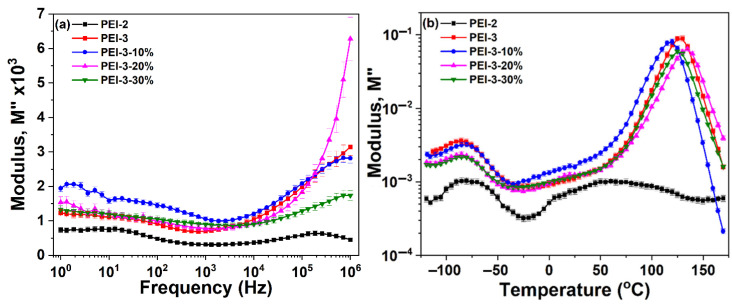
The evolution of modulus with frequency at room temperature (**a**) and with temperature at 10 Hz (**b**) for the studied materials.

Generally, the electrical modulus, *M″*, also shows the dipolar-type signal and conductivity-related signal of electrical charges. At room temperature, the isothermal plots of *M″* as a function of frequency (Figure 12a) showed clearly the signal of the movement of dipoles, with no indication of conductivity. According to Figure 12b, the dominant dielectric peak depicted at temperatures between 50 and 170 °C correlated well with the continuous increase in *ε*″ detected in Figure 11b. In the frame of the latter observation, the dielectric signal observed at high temperatures may be assigned to the transport of free charge through the copolymer lattice.

**Figure 13:**
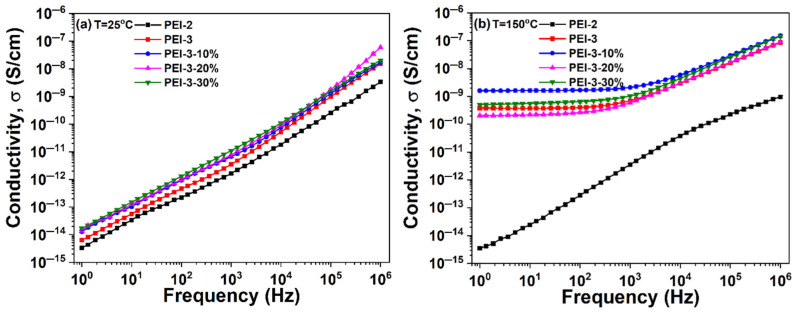
The evolution of measured conductivity with frequency at room temperature (**a**) and at 150 °C (**b**) for the studied materials.

Figure 13 presents the evolution of measured conductivity, *σ*, as a function of frequency at room temperature and at 150 °C, respectively. At room temperature (Figure 13a), a linear increase in *σ* with increasing frequency was detected for all examined samples. This behavior is of the capacitive type and is connected with the movement of dipoles [40]. At a frequency of 1 Hz, the numerical values of *σ* were between 3.3 × 10^−15^ S/cm and 1.7 × 10^−14^ S/cm. On the other hand, at a temperature of 150 °C, the *σ* (T) profiles of Jeffamine-based copolymers enclosed a resistive regime (flat plateau region at low frequencies) and a capacitive regime limited at high frequencies. The resistive behavior is characteristic to the existence of free charges in the material [41]. The *σ* of PEI was 3.5 × 10^−15^ S/cm, while *σ* of copolymers was substantially improved by almost 6 orders of magnitude, up to 1.6 × 10^−9^ S/cm.

## 4. Conclusions

PEI containing Jeffamine segments and composite films based on them and BaTiO_3_ nanoparticles were prepared and characterized by FTIR spectroscopy, SEM, DSC, TG/MS/FTIR, static contact angle, and BDS measurements. A study of the thermal decomposition of these polymers was carried out using TG/MS/FTIR measurements. The influence of the Jeffamine segment content in the polymer structures on the thermal decomposition behavior was determined. The presence of Jeffamine segments in the chemical structure of PEI decreased the thermal stability, especially in oxidative atmosphere. Based on the obtained results, a mechanism of thermal decomposition was proposed, and the main decomposition products were identified. SEM micrographs of the composite films showed that BaTiO_3_ nanoparticles were uniformly distributed in the PEI-3 matrix. The addition of BaTiO_3_ increased the wettability of the surfaces up to 13° for PEI-3-30% containing polar Jeffamine segments and BaTiO_3_ nanoparticles, as determined by measurement of the static contact angle of the film surfaces. The same segment was responsible for the increasing value of *γ_SV_* from 39.48 mN/m in PEI-2 up to 61.42 mN/m in PEI-3, while the introduction of BaTiO_3_ increased *γ_SV_* up to 92.17 mN/m in PEI-3-30% material. Dielectric properties of the composite films, determined by BDS measurements, showed that an increase in the dielectric constant appeared due to the introduction of Jeffamine segments in the chemical structure of PEI and the incorporation of BaTiO_3_ nanoparticles. The samples exhibited a strong increase in dielectric loss and electrical conductivity, especially at temperatures higher than their glass transition temperature; thus, *σ* reached values up to 1.6 × 10^−9^ S/cm in the Jeffamine-containing copolymer and in the subsequent composites containing BaTiO_3_ nanoparticles.

## Figures and Tables

**Table 1:** Thermal characteristics in nitrogen and air and glass transition temperature of the PEI (1–3) and PEI-3-20% composite film.

Sample	T_onset_ (Nitrogen) ^1^ (°C)	T_onset_ (Air) ^1^ (°C)	Char yield in Nitrogen at 650 °C (%)	Char Yield in Air at 650 °C (%)	*T_g_*^2^ (°C)
PEI-1	394	389	16.90	12.22	nd
PEI-2	460	418	53.78	51.85	158
PEI-3	403	371	39.38	26.49	100
PEI-3-20%	395	367	49.16	24.22	96

^1^ Temperature at which the thermal degradation starts; ^2^ Glass transition temperature midpoint.

**Table 2:** Static contact angle values and surface parameters of prepared samples.

Sample	Contact Angle (^o^)		*W_a_* (mN/m)		*γ_SV_* (mN/m)	*γ^p^_SV_* (mN/m)	*γ^d^_SV_* (mN/m)	*γ_SL_* (mN/m)
	W	EG	W	EG				
PEI-2	61.78	43.35	107.22	82.90	39.48	27.62	11.85	5.05
PEI-3	49.89	51.80	119.69	77.68	61.42	60.59	0.83	14.52
PEI-3-10%	62.91	34.18	105.94	87.70	40.42	19.77	20.65	7.27
PEI-3-20%	55.99	33.01	113.51	88.25	44.26	30.27	13.98	3.54
PEI-3-30%	13.37	25.24	143.62	91.41	92.17	91.62	0.54	21.34

## Data Availability

The data presented in this study are available on request from the corresponding author.

## Supplementary Materials

The following supporting information can be downloaded at: https://www.mdpi.com/article/10.3390/polym14214715/s1, Figure S1: 1H NMR spectra of PEI-3; Figure S2: EDX spectra of PEI-3 (a), PEI-3-10% (b), PEI-3-20% (c) and PEI-3-30% (d); Figure S3: TG (a) and DTG (b) curves in nitrogen; Figure S4: TG (a) and DTG (b) in air; Figure S5: DSC curves of the samples; Figure S6: Variation of ion current with temperature for fragments m/z = 18, m/z = 28, m/z = 30 and m/z = 44 if thermal decomposition occurs in nitrogen; Figure S7: Variation of ion current with temperature for fragments m/z = 78, m/z = 91, m/z = 94 and m/z = 108 if thermal decomposition occurs in nitrogen; Figure S8: Variation of ion current with temperature for fragments m/z = 103 for thermal decomposition in nitrogen; Figure S9: Variation of ion current with temperature for fragments m/z = 18, m/z = 28, m/z = 30 and m/z = 44 for thermal decomposition in air; Figure S10: Variation of ion current with temperature for fragments m/z = 78, m/z = 91, m/z = 94 și m/z = 108 for thermal decomposition in air; Figure S11: Variation of ion current by temperature for the fragments m/z = 58, m/z = 73, m/z = 15 and m/z = 46 if thermal decomposition occurs in nitrogen; Figure S12: Variation of ion current with temperature for fragments m/z = 58, m/z = 73, m/z = 15 and m/z = 46 if thermal decomposition occurs in air; Figure S13: Variation of ion current with temperature for fragments m/z = 103 for thermal decomposition in air.
