# Supplementary material for: Tailoring Thermal and Electrical Properties of Jeffamine Segmented Polyetherimide Composite Films Containing BaTiO3 particles
Source: Polymers (Basel). 2022 Nov 3;14(21):4715. doi: 10.3390/polym14214715 (PMC9654722; doi:10.3390/polym14214715)
Supplement: Supplementary file 1 [file polymers-14-04715-s001.zip › polymers-1971806-supplementary.pdf]

polymers MDPI

SUPPORTING INFORMATION

Tailoring Thermal and Electrical Properties of Jeffamine Segmented Polyetherimide Composite Films Containing $BaTiO_3$ particles

Corneliu Hamciuc [1,*], Gabriela Lisa [2], Diana Serbezeanu [1], Luiza Mădălina Grădinaru [1], Mihai Asăndulesa [1], Niță Tudorachi [1] and Tăchiță Vlad-Bubulac [1,*]

[1] "Petru Poni" Institute of Macromolecular Chemistry, Aleea Gr. Ghica Voda, 41A, 700487 Iasi, Romania
[2] Department of Chemical Engineering, Faculty of Chemical Engineering and Environmental Protection, Gheorghe Asachi Technical University of Iasi, Bd. Mangeron 73, 700050 Iasi, Romania
* Correspondence: chamciuc@icmpp.ro (C.H.); tvladb@icmpp.ro (T.V.-B.)

Figure S1. ^1H NMR spectra of PEI-3

Figure S2. EDX spectra of PEI-3 (a), PEI-3-10% (b), PEI-3-20% (c) and PEI-3-30% (d)

Figure S3. TG (a) and DTG (b) curves in nitrogen

Figure S4. TG (a) and DTG (b) in air

Figure S5. DSC curves of the samples

Figure S6. Variation of ion current with temperature for fragments m/z=18, m/z=28, m/z=30 and m/z=44 if thermal decomposition occurs in nitrogen

Figure S7. Variation of ion current with temperature for fragments m/z=78, m/z=91, m/z=94 and m/z=108 if thermal decomposition occurs in nitrogen

Figure S8. Variation of ion current with temperature for fragments m/z=103 for thermal decomposition in nitrogen

Figure S9. Variation of ion current with temperature for fragments m/z=18, m/z=28, m/z=30 and m/z=44 for thermal decomposition in air

Figure S10. Variation of ion current with temperature for fragments m/z=78, m/z=91, m/z=94 și m/z=108 for thermal decomposition in air

Figure S11. Variation of ion current by temperature for the fragments m/z=58, m/z=73, m/z=15 and m/z=46 if thermal decomposition occurs in nitrogen

Figure S12. Variation of ion current with temperature for fragments m/z=58, m/z=73, m/z=15 and m/z=46 if thermal decomposition occurs in air

Figure S13. Variation of ion current with temperature for fragments m/z=103 for thermal decomposition in air

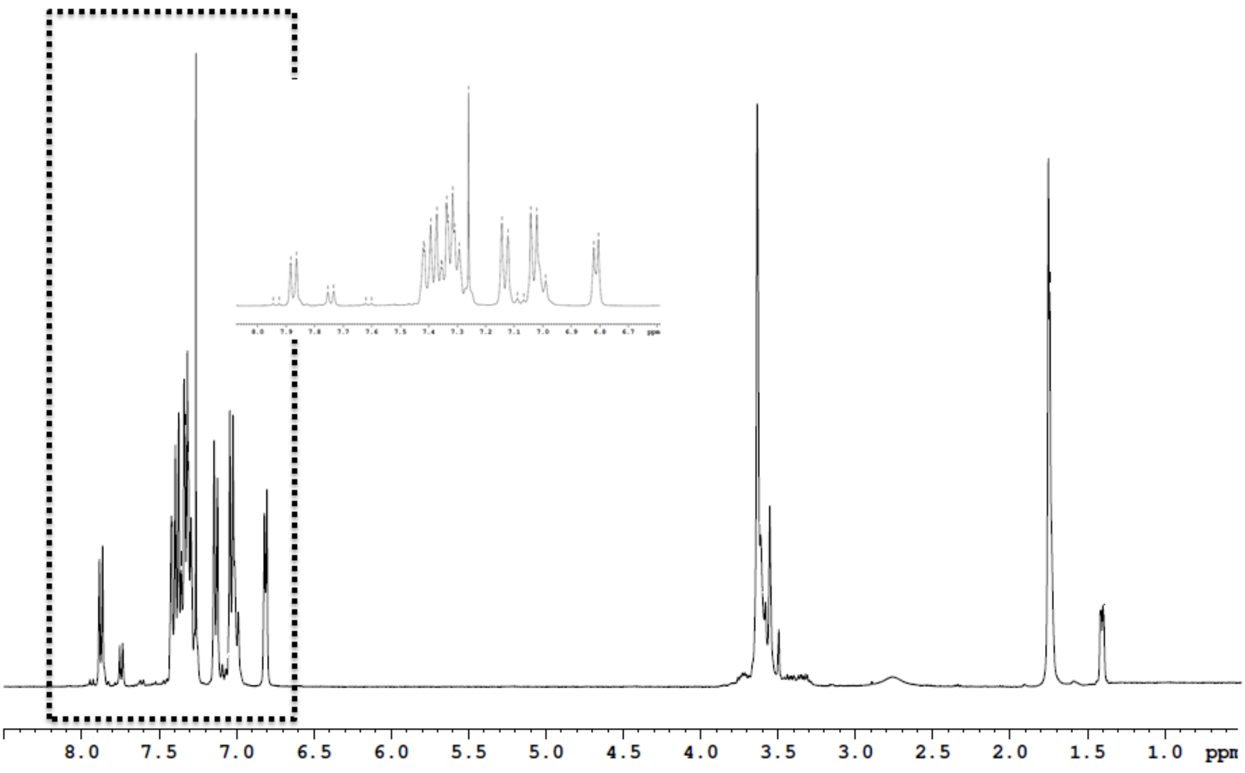

Figure S1. ^{1}H NMR spectra of PEI-3

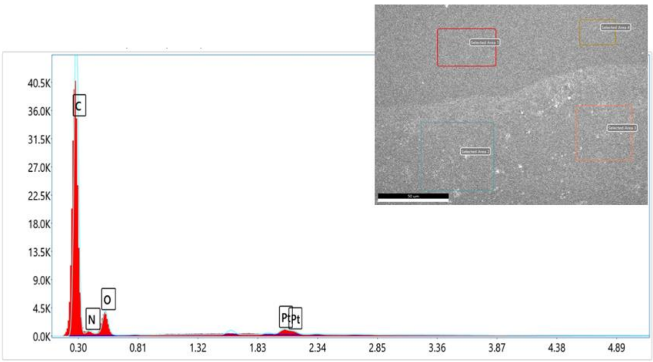

(a)

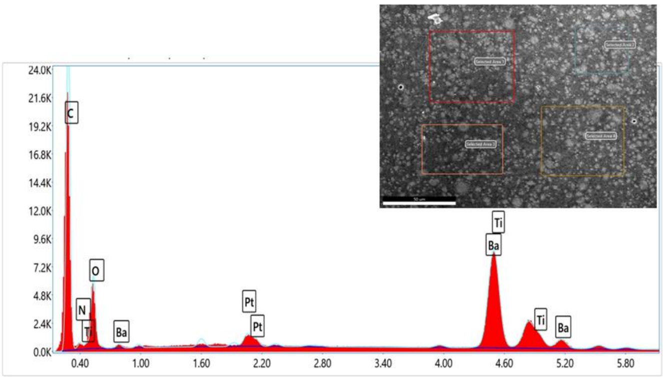

(b)

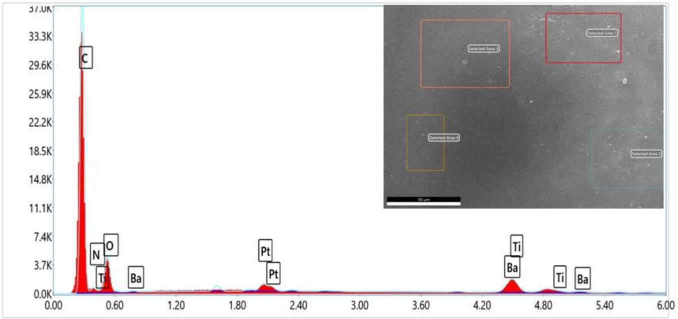

(c)

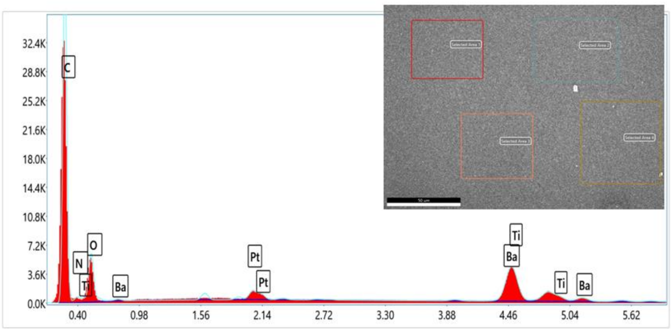

(d)

Figure S2. EDX spectra of PEI-3 (a), PEI-3-10% (b), PEI-3-20% (c) and PEI-3-30% (d)

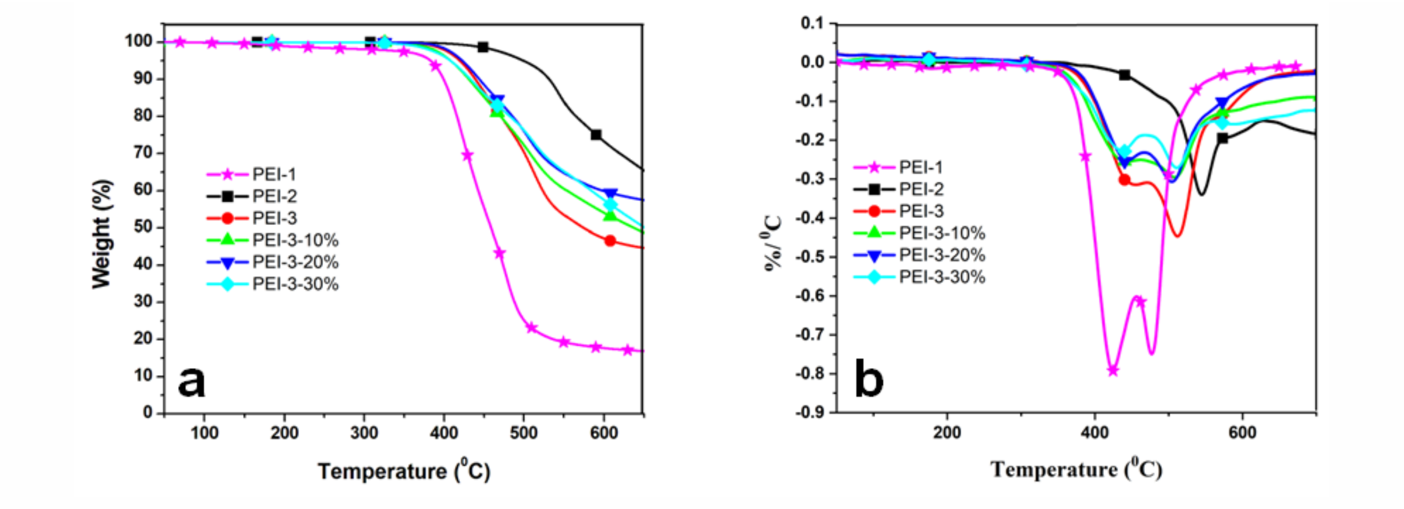

Figure S3. TG (a) and DTG (b) curves in nitrogen

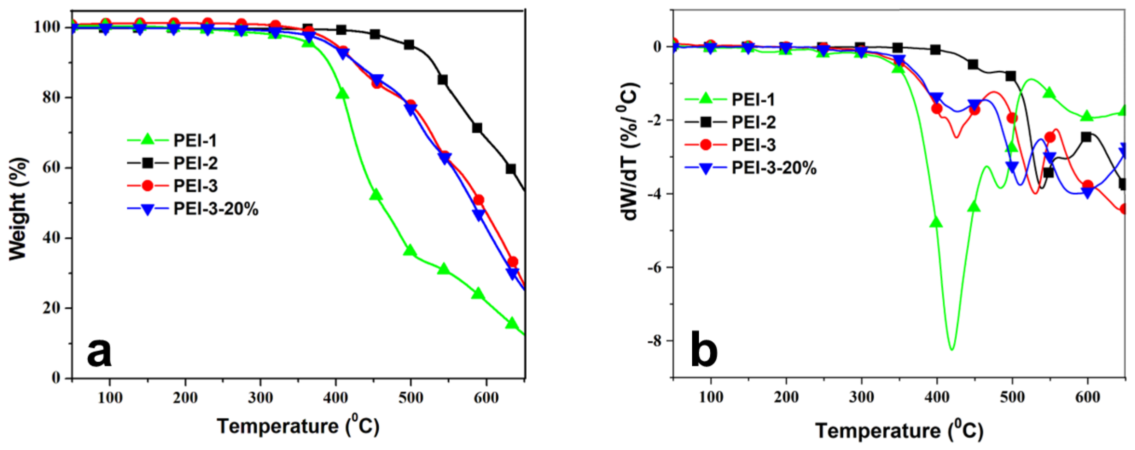

Figure S4. TG (a) and DTG (b) in air.

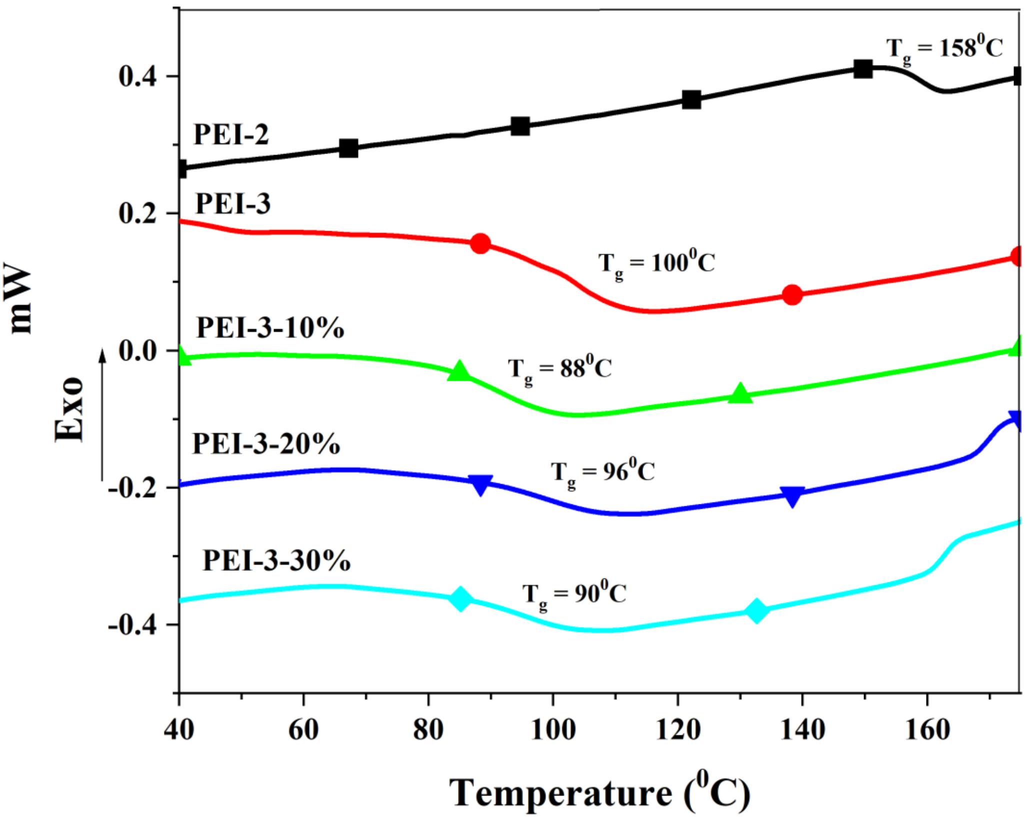

Figure S5. DSC curves of the samples

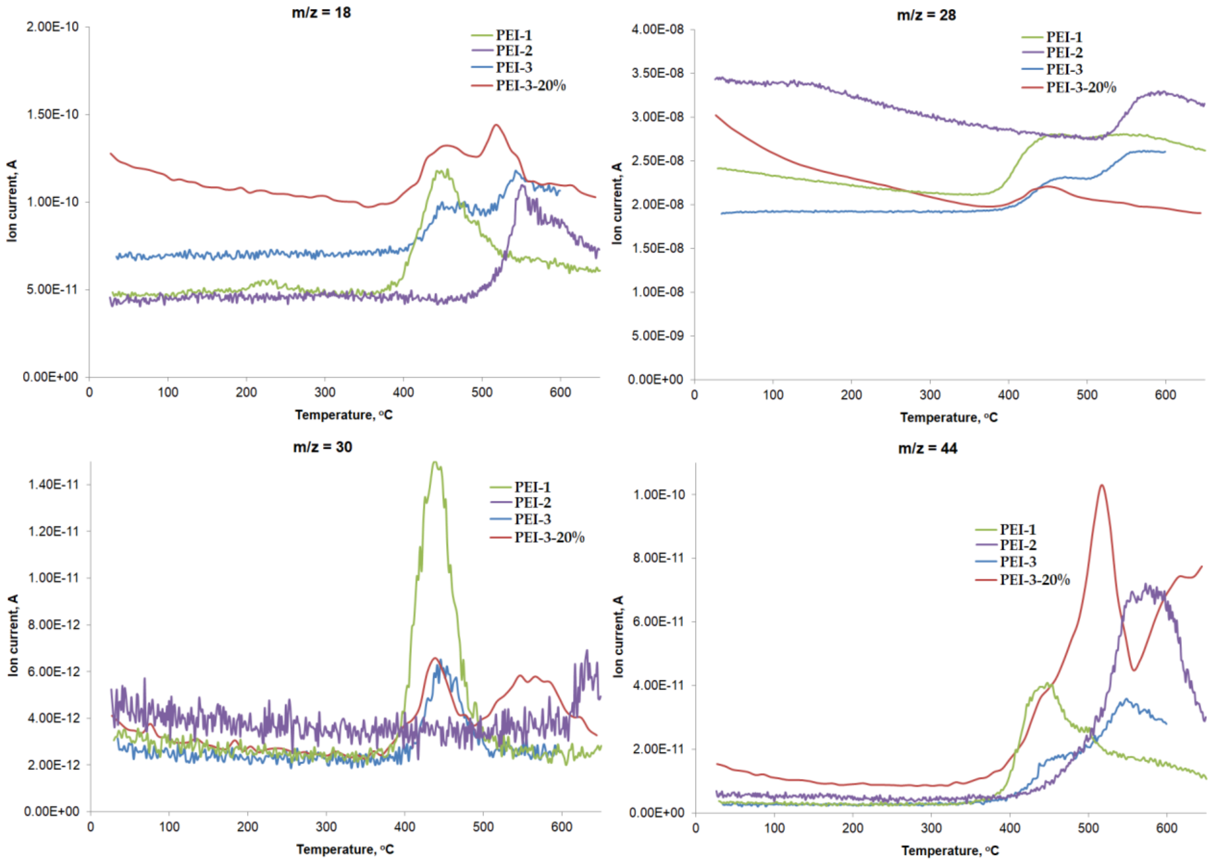

Figure S6. Variation of ion current with temperature for fragments m/z=18, m/z=28, m/z=30 and m/z=44 if thermal decomposition occurs in nitrogen

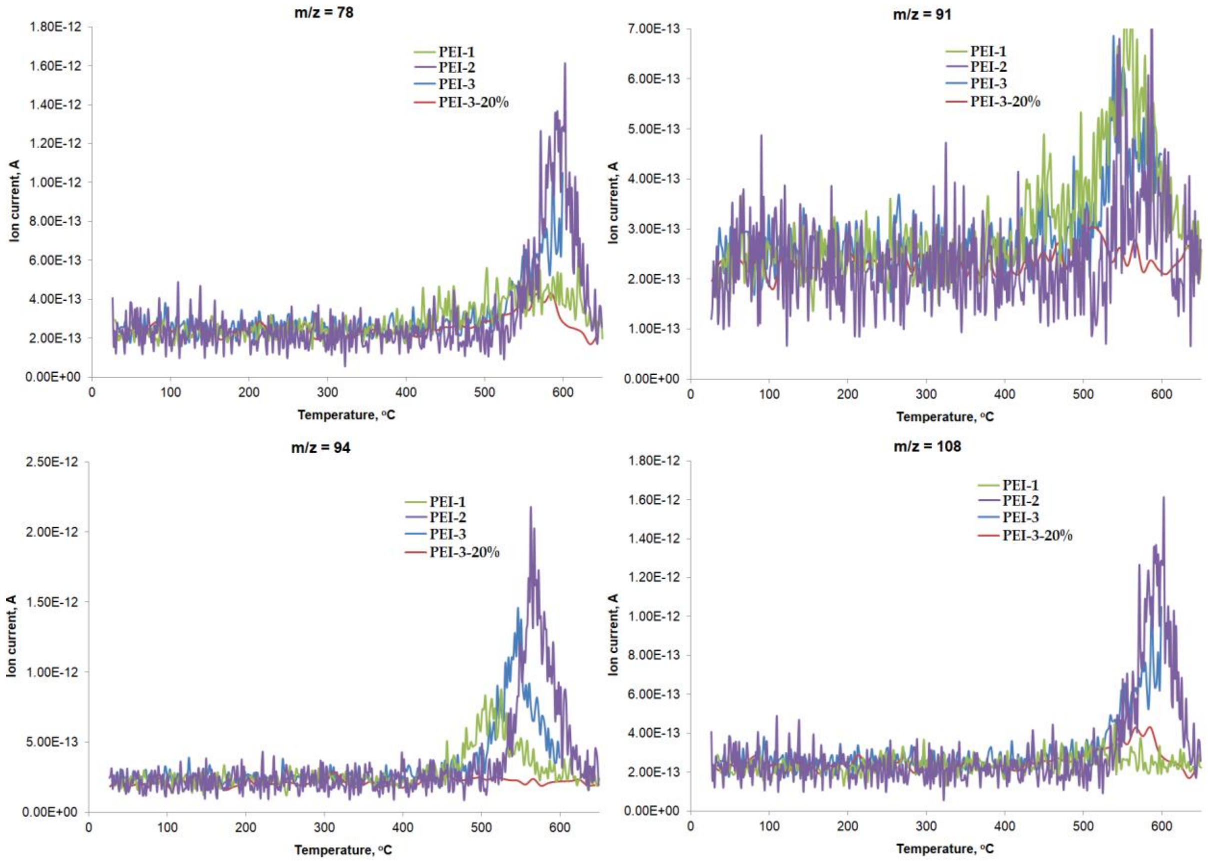

Figure S7. Variation of ion current with temperature for fragments m/z=78, m/z=91, m/z=94 and m/z=108 if thermal decomposition occurs in nitrogen

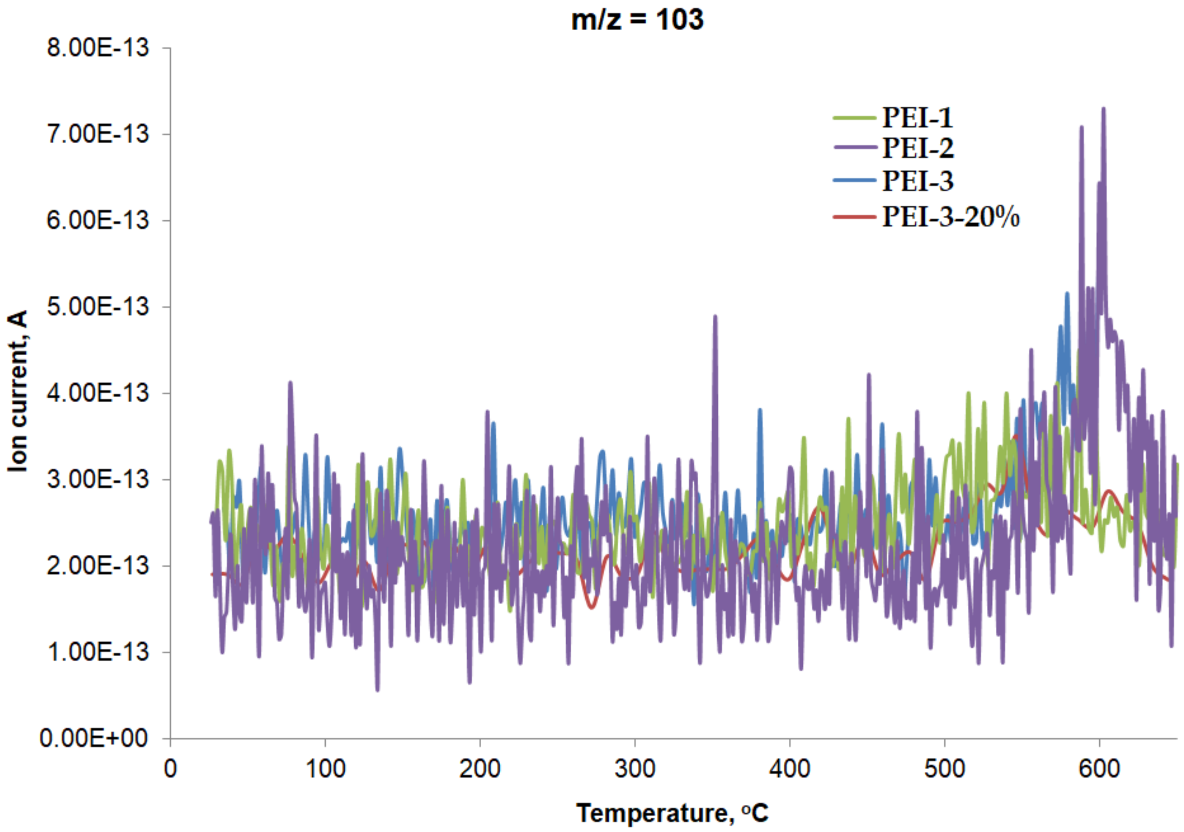

Figure S8. Variation of ion current with temperature for fragments m/z=103for thermal decomposition in nitrogen

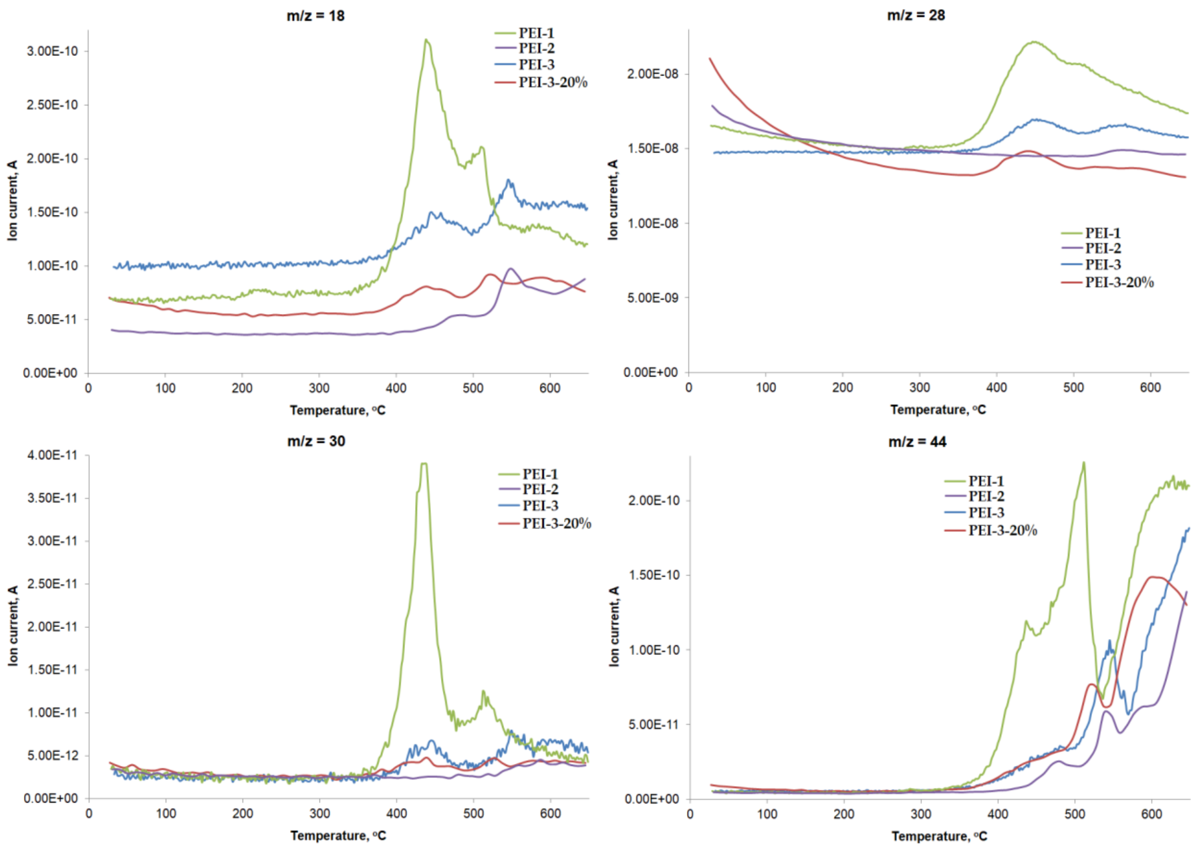

Figure S9. Variation of ion current with temperature for fragments m/z=18, m/z=28, m/z=30 and m/z=44 for thermal decomposition in air

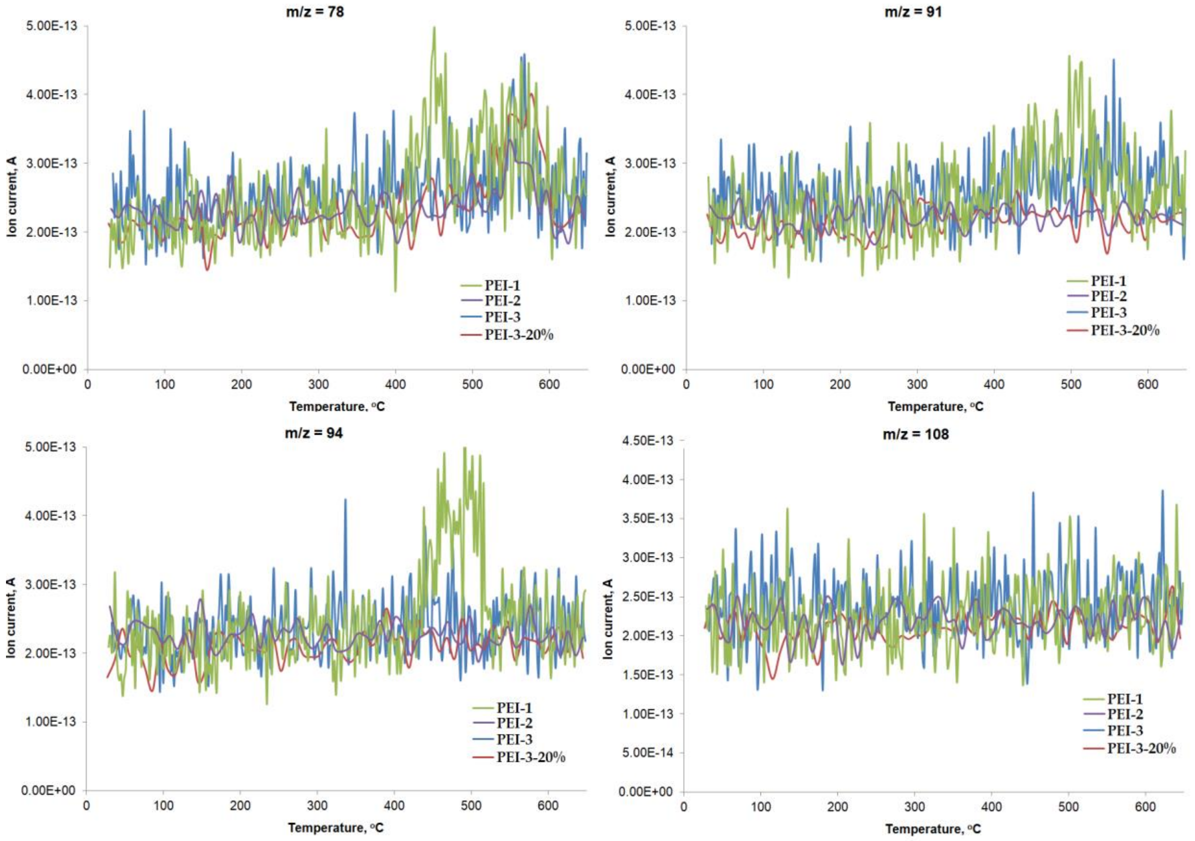

Figure S10. Variation of ion current with temperature for fragments m/z=78, m/z=91, m/z=94 și m/z=108 for thermal decomposition in air

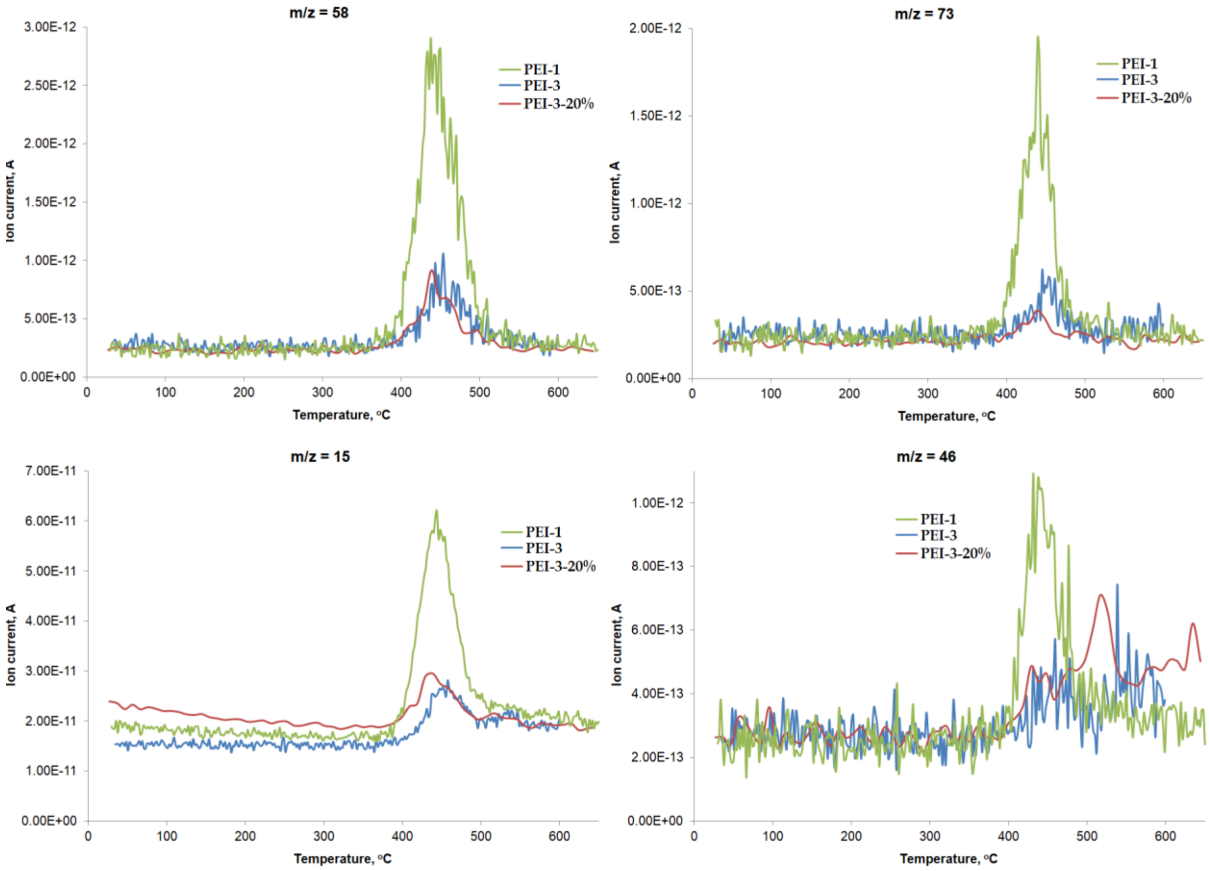

Figure S11. Variation of ion current by temperature for the fragments m/z=58, m/z=73, m/z=15 and m/z=46 if thermal decomposition occurs in nitrogen

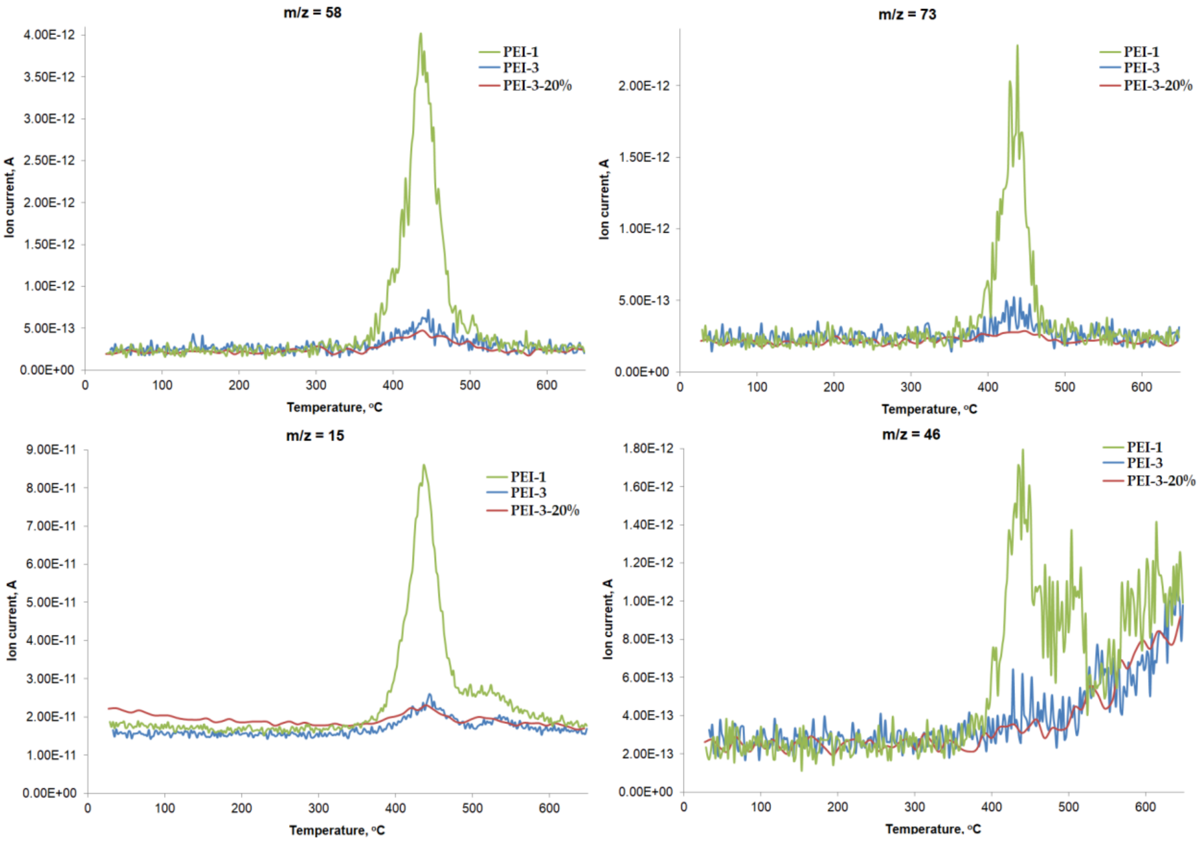

Figure S12. Variation of ion current with temperature for fragments m/z=58, m/z=73, m/z=15 and m/z=46 if thermal decomposition occurs in air.

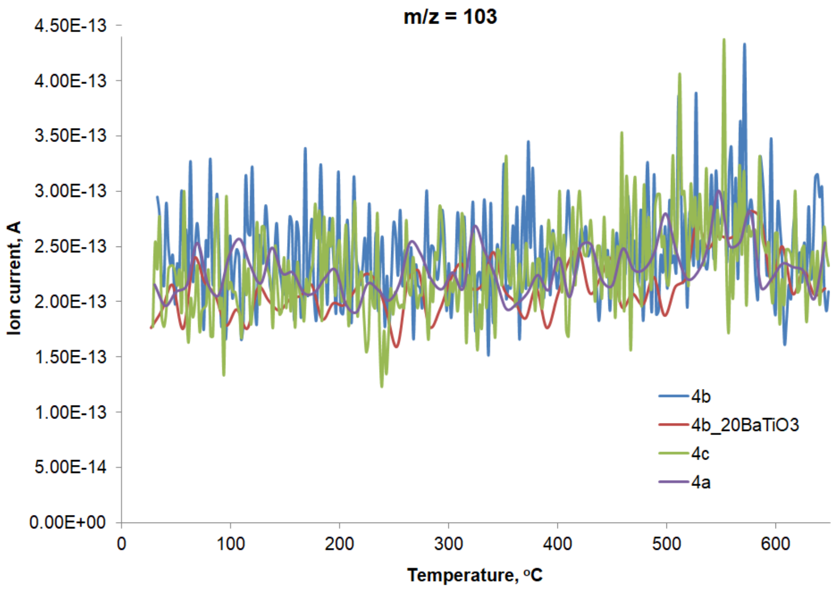

Figure S13. Variation of ion current with temperature for fragments m/z=103 for thermal decomposition in air
